# Radiomics utilization to differentiate nonfunctional adenoma in essential hypertension and functional adenoma in primary aldosteronism

**DOI:** 10.1038/s41598-022-12835-9

**Published:** 2022-05-25

**Authors:** Po-Ting Chen, Dawei Chang, Kao-Lang Liu, Wei-Chih Liao, Weichung Wang, Chin-Chen Chang, Vin-Cent Wu, Yen-Hung Lin

**Affiliations:** 1 Department of Medical Imaging, National Taiwan University Hospital, National Taiwan University College of Medicine, No. 7, Chung-Shan South Road, Taipei, 10002 Taiwan; 2 Institute of Applied Mathematical Sciences, National Taiwan University, Taipei, Taiwan; 3 Department of Medical Imaging, National Taiwan University Cancer Center, Taipei, Taiwan; 4 Division of Gastroenterology and Hepatology, Department of Internal Medicine, National Taiwan University Hospital, National Taiwan University College of Medicine, Taipei, Taiwan; 5 Internal Medicine, College of Medicine, National Taiwan University, Taipei, Taiwan; 6 Department and Graduate Institute of Forensic Medicine, National Taiwan University College of Medicine, Taipei, Taiwan; 7 Division of Nephrology, Department of Internal Medicine, National Taiwan University Hospital, National Taiwan University College of Medicine, Taipei, Taiwan; 8 Division of Cardiology, Department of Internal Medicine, National Taiwan University Hospital, National Taiwan University College of Medicine, Taipei, Taiwan

**Keywords:** Diagnostic markers, Adrenal gland diseases, Machine learning

## Abstract

We performed the present study to investigate the role of computed tomography (CT) radiomics in differentiating nonfunctional adenoma and aldosterone-producing adenoma (APA) and outcome prediction in patients with clinically suspected primary aldosteronism (PA). This study included 60 patients diagnosed with essential hypertension (EH) with nonfunctional adenoma on CT and 91 patients with unilateral surgically proven APA. Each whole nodule on unenhanced and venous phase CT images was segmented manually and randomly split into training and test sets at a ratio of 8:2. Radiomic models for nodule discrimination and outcome prediction of APA after adrenalectomy were established separately using the training set by least absolute shrinkage and selection operator (LASSO) logistic regression, and the performance was evaluated on test sets. The model can differentiate adrenal nodules in EH and PA with a sensitivity, specificity, and accuracy of 83.3%, 78.9% and 80.6% (AUC = 0.91 [0.72, 0.97]) in unenhanced CT and 81.2%, 100% and 87.5% (AUC = 0.98 [0.77, 1.00]) in venous phase CT, respectively. In the outcome after adrenalectomy, the models showed a favorable ability to predict biochemical success (Unenhanced/venous CT: AUC = 0.67 [0.52, 0.79]/0.62 [0.46, 0.76]) and clinical success (Unenhanced/venous CT: AUC = 0.59 [0.47, 0.70]/0.64 [0.51, 0.74]). The results showed that CT-based radiomic models hold promise to discriminate APA and nonfunctional adenoma when an adrenal incidentaloma was detected on CT images of hypertensive patients in clinical practice, while the role of radiomic analysis in outcome prediction after adrenalectomy needs further investigation.

## Introduction

An adrenal incidentaloma is an unexpectedly discovered nodular lesion by radiologic examination, with greater frequency because of the increased use and improved quality of cross-sectional imaging^1^. For patients with hypertension and an adrenal incidentaloma, a further endocrine examination of aldosterone-to-renin activity is suggested to rule out primary aldosteronism (PA), a disorder with excess production of the hormone aldosterone from the adrenal glands, resulting in low renin levels and secondary hypertension^2,3^.

While most of the patients with high blood pressure result from essential hypertension (EH), in which secondary causes are not present, PA is present in 5–13% of hypertensive people^4,5^. PA can be mainly divided into two subtypes: aldosterone-producing adenoma (APA) and idiopathic adrenal hyperplasia (IAH), in which the former can be resected and possibly cured^6^. Computed tomography (CT) is one of the most commonly used imaging modalities for the adrenal gland before surgery. However, there is no suitable imaging tool sufficient to differentiate between aldosterone-producing adenoma and nonfunctional adrenal adenoma. To further determine the lateralization of aldosterone excess and guide the surgery, patients with clinically suspected PA are often recommended for adrenal venous sampling, an invasive procedure requiring experienced operator and fluoroscopic guidance^7^. Aldosterone-producing adenoma can be further diagnosed using CYP11B1/2 immunohistochemistry after surgery, but there are limited laboratories capable of analysis^8^.

Radiomics is an emerging research tool in medical images that enables high-throughput extraction of features representing quantitative information on density, shape, and texture^9^. CT radiomics and texture analysis have been used for studies on distinguishing benign from malignant adrenal nodules^10–12^, differentiating pheochromocytoma from lipid-poor adenoma^13^ and localizing primary aldosteronism^14^. Ascertaining the radiomic analysis may enable detection of subtle differences between aldosterone-producing adenoma and nonfunctional adrenal adenoma, possibly to improve the clinical work-up of adrenal incidentaloma in patients with hypertension. The purpose of the present study was to identify the CT radiomic signature and develop a prediction model to distinguish adrenal adenoma in PA patients using patients with EH as a reference.

## Results

The clinical characteristics of the patients with primary aldosteronism and essential hypertension are summarized in Table 1. No significant differences in sex, systolic/diastolic blood pressure, or duration of hypertension were found between the two cohorts. The patient age in the EH group was older than that in the PA group. In the PA group, the patients showed higher plasma aldosterone concentration (PAC), lower plasma renin activity (PRA), and higher plasma aldosterone to renin ratio (ARR), consistent with the diagnosis of primary aldosteronism. The image density of adrenal nodules in EH was higher than that in PA (median and interquartile range: EH: 5.19 ± 17.48 HU vs. PA: 1.41 ± 15.2 HU; p < 0.05). The long-axis diameter of the largest cross-sectional area was greater in PA (EH: 18.04 ± 8.78 mm vs. PA: 27.36 ± 10.75 mm; p < 0.05), while there was no difference in the volume of nodules between the two groups (EH: 3062.76 ± 7623.38 mm^3^ vs. PA: 4366.18 ± 7155.52 mm^3^; p = 0.294).

**Table 1:** The clinical characteristics of the patients.

Clinical data	EH (n = 56)	PA (n = 89)	p-value
Sex, male (%)	29 (51.7%)	53 (59.5%)	0.358
Age, years	61.2 ± 11.5	52.5 ± 10.8	< 0.01
BMI, kg/m^2^	25.21 ± 4.52	24.26 ± 3.89	0.284
Duration of hypertension, years	4.19 ± 6.87	7.26 ± 7.22	0.059
Systolic blood pressure, mmHg	146.53 ± 23.94	151.33 ± 20.49	0.342
Diastolic blood pressure, mmHg	86.00 ± 14.78	91.57 ± 14.04	0.092
Potassium, mmol/L	4.07 ± 0.42	3.69 ± 0.75	< 0.01
PAC^a^, ng/dL	30.50 (16.74–46.45)	57.15 (33.42–90.05)	< 0.001
PRA^a^, ng/mL/h	1.79 (0.64–5.07)	0.205 (0.078–0.475)	< 0.001
ARR^a^	18.86 (6.61–33.96)	272.64 (65.27–761.59)	< 0.001
eGFR, ml/min/1.73m^2^	90.51 ± 22.52	90.48 ± 23.73	0.995

Data were presented as the mean ± SD, median (interquartile range) or number (%).

*PAC* plasma aldosterone concentration, *PRA* plasma renin activity, *ARR* aldosterone to renin ratio.

^a^Expressed as median and interquartile range.

### Feature selection and radiomics signature construction

The imaging characteristics of adrenal nodules in EH and PA were similar and difficult to differentiate by the naked eye. We extracted image features from the VOIs (volume of interest) of adrenal nodules on unenhanced and venous phase images. Of the 105 radiomics features, the most stable features (46 in unenhanced CT and 45 in venous phase CT) with both intraobserver and interobserver intraclass correlation coefficient (ICC) values > 0.75 were selected for subsequent analysis. After data splitting, the significant features to differentiate adrenal nodules in the training set were selected after LASSO exported the optimal value of the LASSO tuning parameter by using the minimum criteria and the 1 standard error of the minimum criteria (1-SE criteria). Features corresponding to the optimal α were derived, and coefficients were calculated.

**Figure 1:**
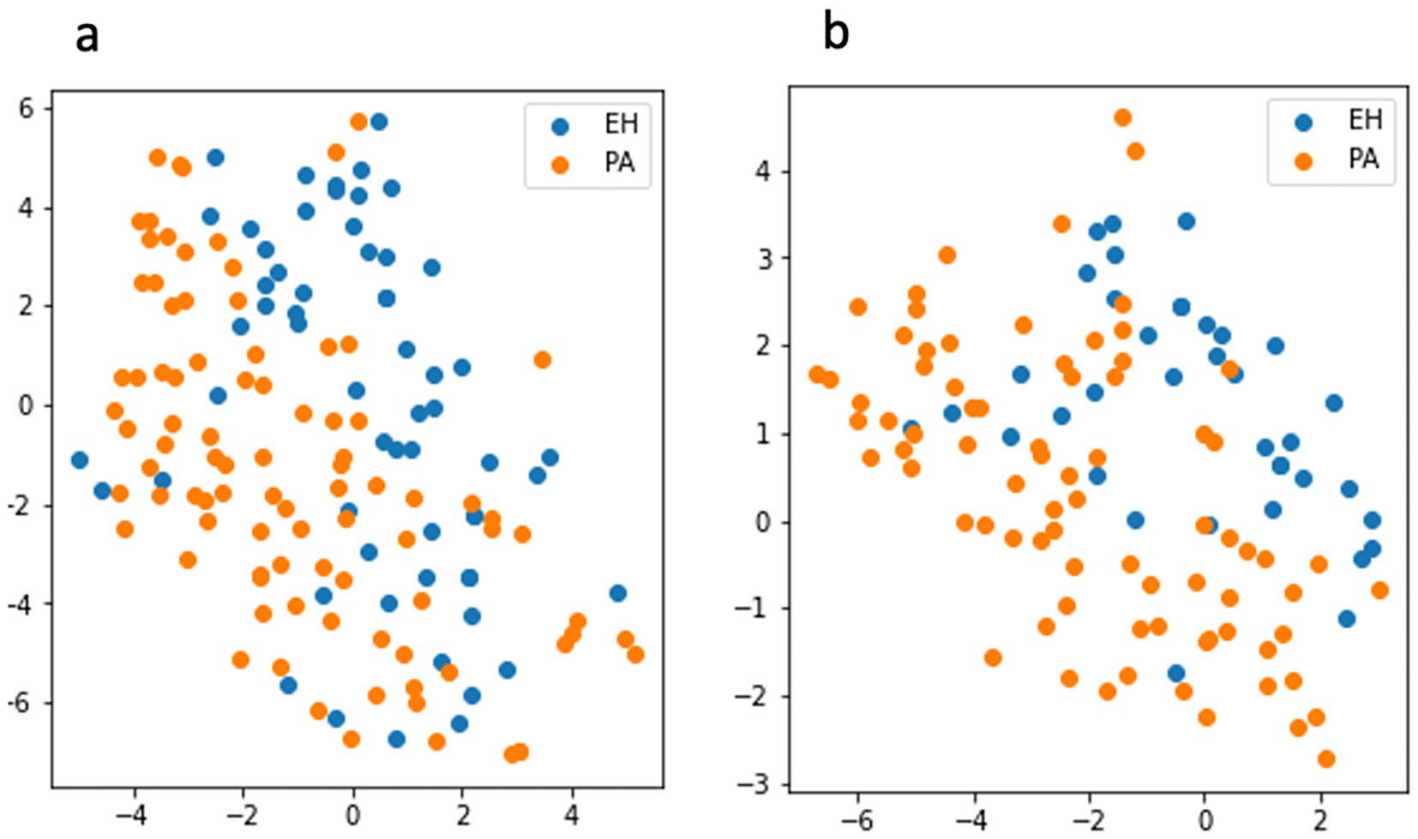
Separation of adrenal nodules in patients with PA and EH by t-distributed stochastic neighbor embedding (t-SNE). A two-dimensional scatter plot via t-SNE visualization (perplexity = 50) was based on the features selected in unenhanced CT (**a**) and venous phase CT (**b**).

On unenhanced CT, there were a total of 91 PA nodules and 60 EH nodules included. The data were randomly split into a training set (72 PA/48 EH) and a test set (19 PA/12 EH). Eight features were selected from the training set after LASSO regression (two first-order statistical features, one shape feature, and five textural features [GLCM n = 1; GLDM n = 2; GLSZM n = 2]). In venous phase contrast-enhanced CT, there were a total of 79 PA nodules and 37 EH nodules that were randomly split into a training set (63PA/29EH) and test set (16PA/8EH). A total of 16 features were selected from the training set after LASSO regression (two first-order statistical features, two shape features and twelve textural features [GLCM n = 5; GLDM n = 3; GLSZM n = 1; GLRLM n = 2; NGTDM n = 1]). The selected differential radiomic features and their coefficients on unenhanced and venous phase contrast-enhanced CT are summarized in Table 2. A t-distributed stochastic neighbor embedding (t-SNE) method^15^ based on features selected in unenhanced and venous CT to visualize how well these features separate PA nodules and EH nodules (Fig. 1).

**Table 2:** Selected radiomic features in unenhanced and venous phase contrast-enhanced CT.

Images	Features	Coefficient	Mean values	Standard deviation
Unenhanced	Gldm: dependence variance	− 1.536	8.505	3.179
	Shape: minor axis length	− 1.3	14.224	6.286
	Glszm: large area low gray level emphasis	1.246	92.626	180.152
	Glcm: joint energy	0.855	0.039	0.016
	Firstorder: robust mean absolute deviation	− 0.663	15.17	4.222
	Gldm: small dependence emphasis	− 0.286	0.133	0.049
	Glszm: large area emphasis	0.165	6895.076	17,545.511
	Firstorder: mean	0.032	4.817	13.971
Venous phase	Glrlm: short run emphasis	− 6.127	0.874	0.033
	Glcm: inverse variance	− 5.867	0.424	0.043
	Glrlm: run length non uniformity normalized	− 4.762	0.725	0.06
	Gldm: dependence non uniformity normalized	4.585	0.11	0.028
	Firstorder: uniformity	4.35	0.16	0.039
	Ngtdm: strength	3.139	0.733	0.672
	Gldm: small dependence emphasis	− 2.896	0.146	0.054
	Gldm: dependence variance	− 2.471	8.072	3.039
	Glcm: maximum probability	− 2.061	0.071	0.032
	Glcm: Idn	− 2.042	0.908	0.017
	Glcm: difference variance	− 1.63	1.986	0.771
	Shape: maximum 2D diameter column	− 1.263	18.0643	7.888
	Shape: least axis length	0.862	10.956	5.604
	Glcm: Idmn	− 0.739	0.98	0.007
	Glszm: large area high gray level emphasis	0.414	716,436.296	3,263,740.816
	Firstorder: interquartile range	− 0.045	39.764	11.32

### Radiomic model in unenhanced and venous phase CT

**Figure 2:**
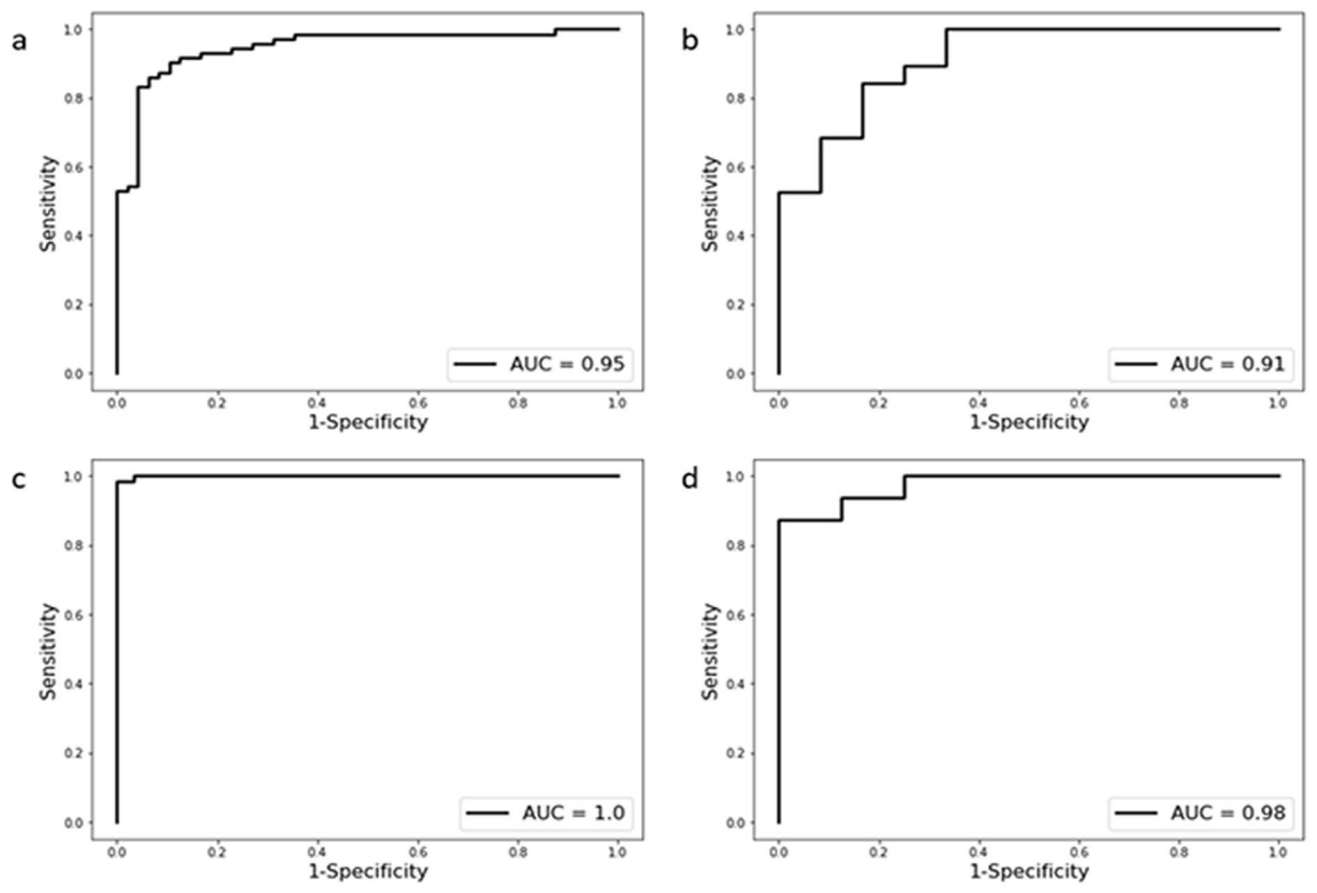
Performance of radiomic model in unenhanced and venous phase CT. Receiver operating characteristic curves of the unenhanced CT radiomic model in the training set (**a**)/test set (**b**) and venous phase CT radiomic model in the training set (**c**)/test set (**d**).

A radiomics-based model was built according to the selected significant radiomic features. In unenhanced CT, the model was calculated using the normalized value of selected features and determined as follows: Logit = 0.122 − 1.536 × GLDM_Dependence Variance − 1.3 × Shape_Minor Axis Length + 1.246 × GLSZM_Large Area Low Gray Level Emphasis + 0.855 × GLCM_Joint Energy – 0.663 × Robust Mean Absolute Deviation − 0.286 × GLDM_Small Dependence Emphasis + 0.165 × GLSZM_Large Area Emphasis + 0.032 × Mean. Using a cutoff value of 0.503 in the unenhanced CT model, the sensitivity, specificity, and accuracy of the model to differentiate PA from EH were 93.7%, 84.7%, and 88.3% (AUC = 0.95 [0.89, 0.98]) in the training set and 83.3%, 78.9%, and 80.6% (AUC = 0.91 [0.72, 0.97]) in the test set. In venous phase CT, the model was also determined using the selected radiomic features. After normalization of the features, the model was constructed using the normalized value and its corresponding coefficient. Using a cutoff of 0.577, the sensitivity, specificity, and accuracy of the model to differentiate PA from EH were 96.8%, 100%, and 97.8% (AUC = 1.00 [0.95, 1.00]) in the training set and 81.2%, 100%, and 87.5% (AUC = 0.98[0.77, 1.00]) in the test set (Fig. 2).

### Radiomic analysis for outcome prediction of APA after adrenalectomy

On unenhanced CT, 73 of 91 patients and 49 of 91 patients achieved biochemical success and clinical success after adrenalectomy, respectively. The sensitivity, specificity, and accuracy were 73.9%, 64.7%, and 72.2% (AUC = 0.67 [0.52, 0.79]) in the radiomic model to predict biochemical success and 89.7%, 34.1%, and 62% (AUC = 0.59 [0.47, 0.70]) in the model to predict clinical success. On venous phase CT, 64 of 79 patients and 43 of 79 patients achieved biomedical success and clinical success after adrenalectomy, respectively. The sensitivity, specificity, and accuracy were 71.9%, 60%, and 88.5% (AUC = 0.62 [0.46, 0.76]) in the model to predict biochemical success and 88.4%, 44.4%, and 65.5% (AUC = 0.64 [0.51, 0.74]) in the model to predict clinical success.

## Discussion

This study identified differentiating radiomic features and showed that radiomics-based analysis could accurately distinguish adrenal nodules in patients with PA and EH in either unenhanced or contrast-enhanced venous phase CT. We also built a radiomic model to further predict the outcome in patients with APA after adrenalectomy. The results provided proof of concept that CT radiomic analysis has the potential for prognosis prediction and clinical treatment application in patients with hypertension, especially in the groups of clinically suspected PA.

When PA is suspected in a patient with hypertension, a plasma aldosterone to renin ratio (ARR) will be obtained initially. Confirmatory laboratory tests are then used for diagnosis in patients with abnormal ARR^5^. The role of abdominal CT is for subgroup evaluation and to exclude malignant tumors, while localization of the source of aldosterone excess in PA is poor on cross-sectional adrenal images^16^. A recent study investigated the usefulness of CT radiomics in predicting the localization of PA with an accuracy of 67%, greater than previous studies using traditional image analysis methods^14^. The results implied that there may be some correlation between texture features and the aldosterone production status of the adrenal gland. Discrimination between aldosterone-producing adenoma and nonfunctional adrenal adenoma on axial imaging with traditional practice of visual interpretation remains challenging, while radiomic analysis provide a more objective way than radiological analysis using naked eyes. In this study, we used the essential hypertensive patients with nonfunctional adenoma as the reference instead of IAH because the functional status of individual adrenocortical tumor on CT is hard to be confirmed in patients with IAH. We identified 8 features on unenhanced CT and 16 features on contrast-enhanced CT, providing classification models with adequate performance to differentiate adrenal nodules in EH and PA patients. In clinical practice, the results provided a tool for radiologists to distinguish aldosterone-producing adenoma and nonfunctional adenoma when an adrenal incidentaloma was detected in CT images of hypertensive patients.

The clinical outcomes of adrenalectomy for unilateral APA patients have previously been investigated using several presurgical clinical factors^17,18^. Our previous study used nonstimulated AVS indices, including the lateralization index and contralateral suppression, to serve as reliable preoperative determinants for predicting the outcomes of adrenalectomy in patients with unilateral PA^19^. In the present study, radiomic analysis achieved modest performance in predicting the outcome in patients with APA after adrenalectomy. Although the performance of radiomic model is not comparable to previous studies using clinical factors, our explorative analysis provides the possibility for further investigation from the imaging perspective.

Radiomics can be used to capture tissue and lesion characteristics extracted from images for further analysis. First-order features are based on the first-order histogram that describes the distribution of voxel intensities within an image. Shape features are used in many settings to define the three-dimensional shape and size of the VOI. Textural features are calculated with GLCM, which describes the spatial distribution of gray level intensities within an image; GLSZM, which indicates the randomness of gray levels in a region by quantifying gray level zones; GLRLM, which calculates the number of contiguous voxels that have the same gray level value and characterizes the gray-level run lengths of different gray-level intensities in any direction; NGTDM, which quantifies the difference between a gray value and the average gray value of its neighbors within a fixed distance; and GLDM, quantifies gray level dependencies in an image^20^. In the selected features of our study, there were five texture features in unenhanced CT and 12 texture features in venous phase CT. The feature with the highest coefficient is gldm_DependenceVariance in unenhanced CT and glrlm_ short-run emphasis in venous phase CT. These texture features describe the spatial distribution of voxel densities within the ROI, which represent lesion heterogeneity and are difficult to perceive by the human eye. These results are comparable to those of several studies on adrenal lesion differentiation. Zhang et al. used CT texture analysis and found that pheochromocytoma was more heterogeneous than lipid-poor adrenal cortical adenoma from a texture perspective^21^. Ho et al. identified several second-order texture features and concluded that increased tumor heterogeneity was the most likely reason for the ability of texture analysis to predict adrenal malignancy on contrast-enhanced CT^11^. While radiomic analysis enables the detection of subtle differences in nonfunctional adenoma and APA, the explanation of complex radiomic features and the direct connection to clinical and histologic representation still require further research.

In our study, radiomic analysis was performed on both unenhanced and venous phase contrast-enhanced images. While there is no significant difference in performance, the selected features in the two models are different. The impact of contrast medium on radiomic feature stability has been studied in oncology patients^22–24^, but there is a limited amount of data in the literature on radiomic analysis of benign adrenal lesions. Adenoma and nonadenoma can be differentiated using the adrenal washout CT protocol, which consists of a noncontrast, contrast-enhanced scan with a delay of 60–90 s and a delayed scan at 10–15 min^25^. The enhancing pattern might facilitate adrenal lesion characterization, and it was speculated that a combination of radiomic features in different phases might improve the performance. In our study, the analysis using a combination of different phase images is not performed because not all the cases have images in both phases.

The challenge of applying radiomic analysis in adrenal incidentaloma is that the nodules are usually small compared to other visceral organs. To provide more data and information, 3D volumetric radiomic analysis under thin-slice (1 mm) reconstruction was used in this study. Shape features could be calculated more precisely in 3D analysis for nodular lesions, but it requires extra time and labor. Until now, there has been no consensus on the best method in radiomic analysis among 3D and 2D approaches^26^. Yi et al. defined a radiomic signature for preoperative differentiation between subclinical pheochromocytoma and lipid-poor adrenal adenoma using the largest cross-sectional area of the tumor^27^. A.A. Ahmed et al. used 3D radiomic features in preoperative CT studies to predict the Ki-67 index in patients with adrenocortical carcinoma^28^. While it is more convenient to use 2D images in clinical applications for radiomic analysis, lesion heterogeneity cannot be expressed in only a single cross-sectional image. Further studies are warranted to compare the costs and benefits in the future.

The current study had some limitations. First, the cases included in this study were based on single-center data, which was relatively small and possibly led to overfitting. To further improve reproducibility, more data across machines and centers are important for the validation of radiomic analysis. Second, the pathology and immunohistochemistry results of adrenal nodules were not confirmed in the group of patients with essential hypertension, which could possibly lead to data heterogeneity. However, it closely resembles the real clinical scenario in which patients with benign lesions will not undergo surgery. Third, manual nodule segmentation is time-consuming, especially in 3D analysis, and it may encounter difficulties in direct clinical application. Further development of an automatic segmentation algorithm for adrenal glands and nodules is warranted.

In conclusion, the present study provides a potential way to use radiomic analysis to differentiate functional adenoma in patients with clinically suspected PA. The analysis can remind clinicians of the necessity of further examination when adrenal incidentalomas were detected in hypertensive patients. CT-based radiomic analysis could also provide a noninvasive method for outcome prediction of APA after adrenalectomy.

## Methods

This was a retrospective analysis of a prospectively collected database. We enrolled patients diagnosed with EH and PA from the Taiwan Primary Aldosteronism Investigation (TAIPAI) database from 2014 to 2017^29^. The study was approved by the Institute Research Ethical Committee of National Taiwan University Hospital (NTUH), which waived the requirement for informed consent from individual patients. All experiments were performed in accordance with the relevant guidelines and regulations.

### Patient characteristics

**Figure 3:**
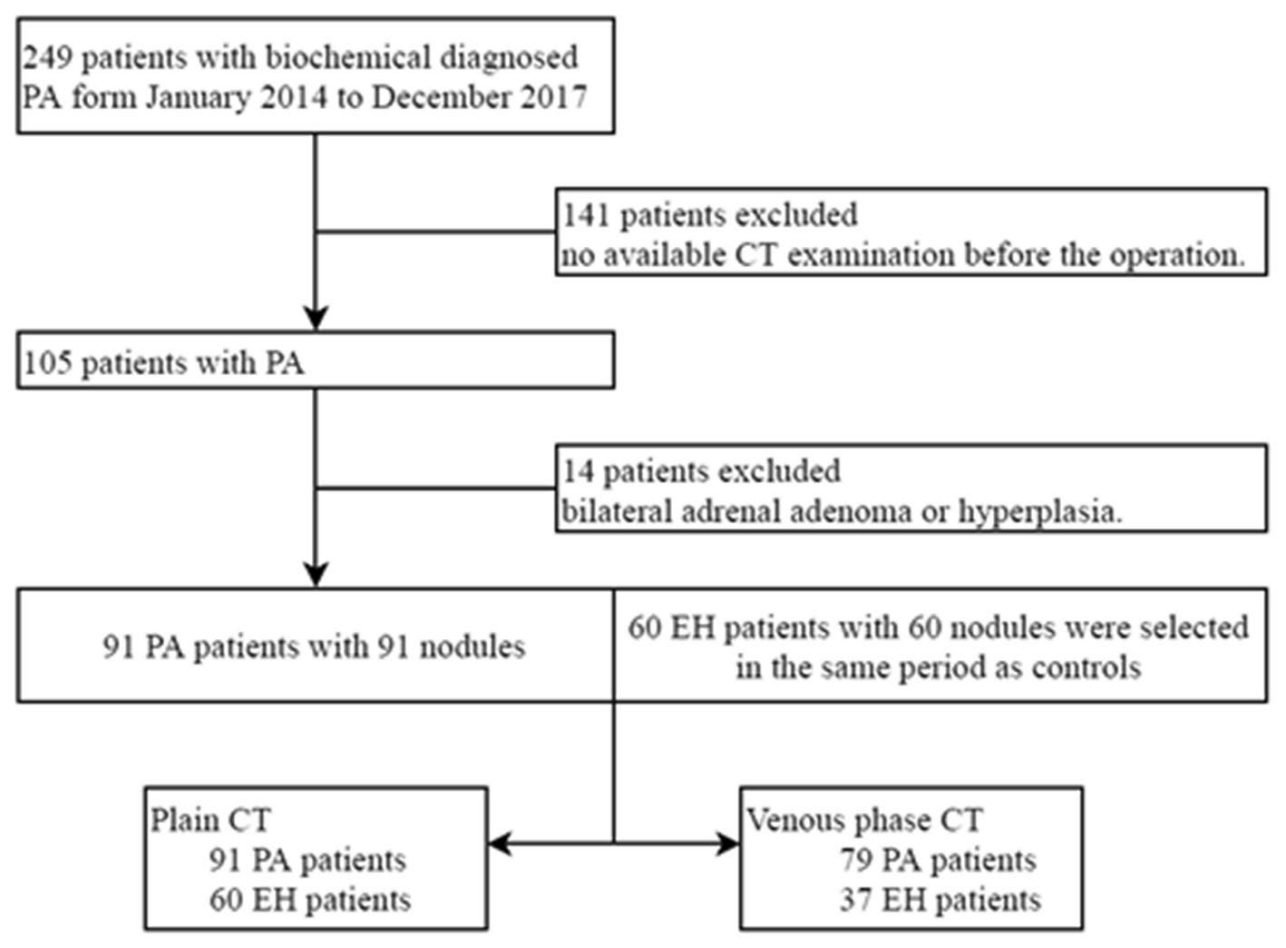
Flow diagram of datasets.

The study population initially consisted of 249 patients with biochemically diagnosed PA who underwent adrenalectomy between January 2014 and December 2017. A total of 141 patients were excluded because there was no available CT examination before the operation. Fourteen patients were excluded due to bilateral adrenal adenoma or hyperplasia. Another 60 patients with EH showing a single adrenal nodule on abdominal CT were included after reviewing our electronic medical and database in the same time period. There were 23 patients with EH and 12 patients with PA who received only unenhanced CT. The numbers of patients and images included in this study are summarized in Fig. 3.

### Diagnosis and classification of PA

PA was diagnosed based on the following criteria: (1) autonomous excess aldosterone production evidenced with an ARR (aldosterone to renin ratio) > 35; (2) a TAIPAI score > 60%^29^ and (3) postsaline loading PAC (plasma aldosterone concentration) > 10 ng/dL, ARR > 35 in a postcaptopril/losartan test, or PAC > 6 ng/dL indicated by a fludrocortisone suppression test.

The classification of PA into subtypes was based on TAIAPI experience^5,30^. IAH was diagnosed according to the following criteria: (1) evidence of bilateral diffuse enlargement on preoperative CT, (2) nonlateralization of aldosterone secretion during adrenal venous sampling, and (3) diffuse cell hyperplasia on biopsy of resected specimens in patients who underwent an operation. APA was diagnosed based on the following criteria: (1) evidence of adenoma on preoperative CT, (2) lateralization of aldosterone secretion during adrenal venous sampling, and (3) pathologically proven adenoma after adrenalectomy with a subsequent cure of hypertension not requiring antihypertensive agents or improvement in hypertension, potassium, PAC, and plasma renin activity (PRA)^31^.

### Outcome analysis after adrenalectomy

The outcomes of patients with APA after adrenalectomy were evaluated according to the criteria established by the PASO consensus^32^. Briefly, clinical success was defined as normal blood pressure without the use of antihypertensive drugs, while biochemical success was evaluated using postoperative serum potassium level and ARR. The outcome analysis for patients with PA was based on clinical or biochemical success.

### CT Image acquisition and adrenal nodule segmentation

CT examinations were performed using one of six CT scanners (Brilliance iCT 256, Philips Healthcare; Sensation 64 and SOMATOM Definition AS + , Siemens Healthcare; Aquilion One, Toshiba; Revolution CT and LightSpeed VCT, GE Healthcare) with 100, 120, or 130 kV automatic mA control and without extra noise reduction processes. The slice thickness ranged between 0.7 and 1.5 mm, and the image size was 512 × 512 pixels. Unenhanced and venous phase CT images were used in the analyses, and all images were reconstructed into 1-mm slices. Venous-phase images were obtained at 70–80 s after intravenous administration of contrast medium (1.5 mL per mL/kg body weight, with an upper limit of 150 mL).

**Figure 4:**
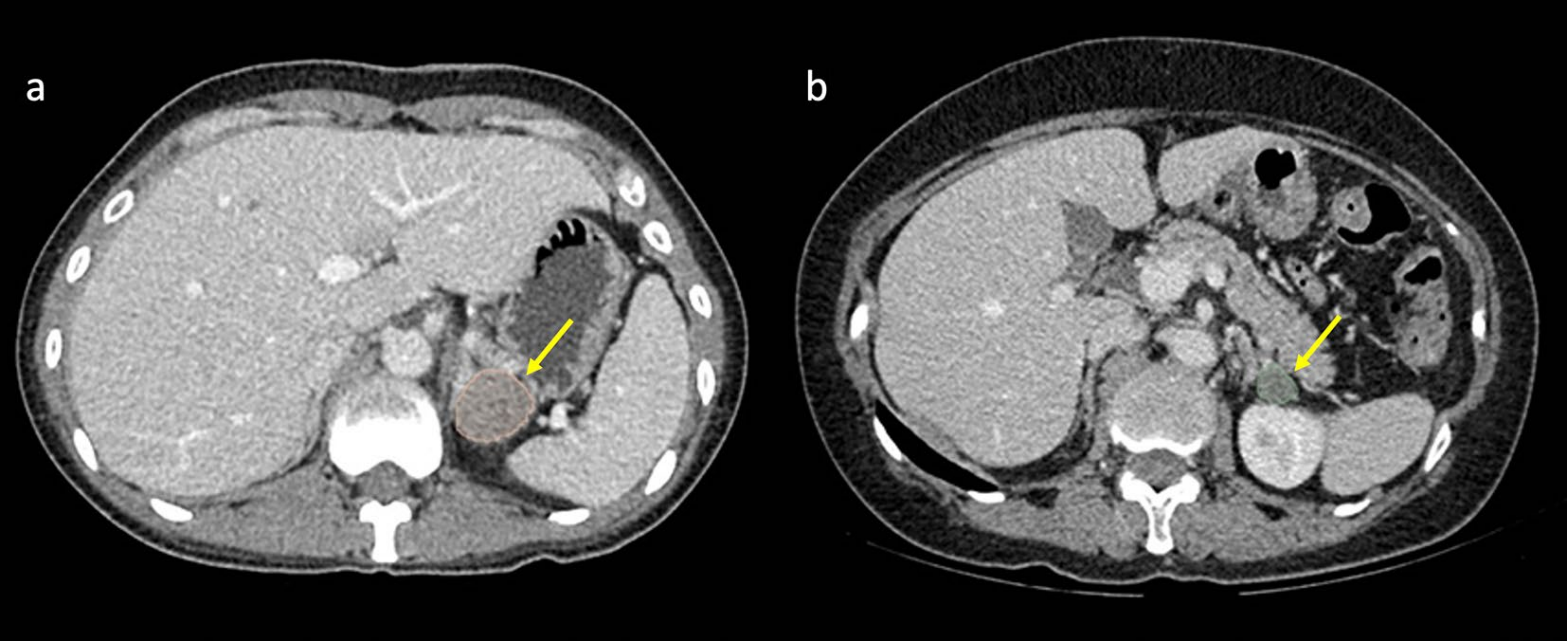
Adrenal nodule segmentation. (**a**) A 38-year-old female with primary aldosteronism refractory to medical treatment for hypertension. Abdominal CT showed a left adrenal nodule (arrow), and clinical success was achieved after laparoscopic adrenalectomy. (**b**) A 64-year-old female diagnosed with essential hypertension with a left adrenal nodule (arrow).

Each whole nodule was segmented on unenhanced and venous phase CT images with soft tissue (width, 1500 HU; level, − 500 HU) window settings. The images were independently segmented manually by two experienced abdominal radiologists (5 years and 15 years of experience) using the open-source software 3D Slicer (version 4.8.1; www. slicer.org) (Fig. 4).

### Radiomic feature extraction and selection

We followed the image biomarker standardization initiative (IBSI) guidelines in conducting the radiomic analysis. The whole adrenal nodules in each CT examination served as VOIs, from which 3D radiomic features were extracted using the open-source platform PyRadiomics. HU values were used for radiomic feature extraction. The CT images were reconstructed to 1 × 1 × 1 mm by linear interpolation before extraction. All 105 nonfiltered features, including 14 shape features, 18 intensity histogram features, and 73 texture features, were extracted for further analysis. The bin size was set to 16 to compute the texture features.

To evaluate the intra- and interobserver reliability of radiomic features, 10 EH and 10 PA patients were randomly selected for ROI segmentation in unenhanced and venous phase images. ROI segmentations were performed in a blind fashion by two radiologists (P.T.C. and C.C.C.) and reanalyzed by a primary radiologist (P.T.C.) 3 months after the first assessment. The intraclass correlation coefficient (ICC) was calculated for reproducibility evaluation. Only the features with both intra- and interobserver ICC values greater than 0.75 that indicated good reliability were selected for further analysis^33^.

### Statistical analyses

Continuous variables were compared using the t-test or Mann–Whitney U test, and categorical variables were compared using the chi-square test or Fisher’s exact test, when appropriate. P values of < 0.05 were considered statistically significant.

The dataset was randomly split into training and test sets at a ratio of 8:2. The classification model was built using a training set based on logistic regression with regularization. First, features from the training set were normalized, and the important and robust features were selected by least absolute shrinkage and selection operator (LASSO) logistic regression, with penalty parameter tuning conducted by fivefold cross-validation. Then, the classification model was built by LASSO logistic regression with the optimal penalty parameter. Model performance was evaluated in the test set. The performance indicators for each prediction model candidate included AUC (area under the curve), accuracy, sensitivity, and specificity. The cutoff point for the final classification model was set using the threshold with the greatest Youden index (sensitivity + specificity -1). To further analyze the outcome in patients with APA, a radiomic model was built in the same way to predict biochemical or clinical success after adrenalectomy. No independent test set was used for this explorative analysis because of small size of dataset for outcome prediction. The calibration curve and the Hosmer–Lemeshow test are performed, and details are provided in the supplementary information S1.

## Supplementary Information

Supplementary Information.
