# Supplementary material for: Radiomics utilization to differentiate nonfunctional adenoma in essential hypertension and functional adenoma in primary aldosteronism
Source: Sci Rep. 2022 May 25;12:8892. doi: 10.1038/s41598-022-12835-9 (PMC9132956; doi:10.1038/s41598-022-12835-9)
Supplement: Supplementary file 1 — Supplementary Information. [file 41598_2022_12835_MOESM1_ESM.docx]

**Supplementary information**

**Calibration curves and Hosmer–Lemeshow test for radiomic models**

Calibration curves were plotted to assess the calibration of the radiomics models accompanied with the Hosmer-Lemeshow test (p > 0.05 indicating good fit). The calibration curve of models for nodule discrimitation (Figure 1) and outcome prediction of aldotersone producing adenoma (APA ) after adrenalectomy (Figures 2) are presented as below.

The Hosmer–Lemeshow test yielded a nonsignificant statistic of test set (p = 0.16) in radiomic model to differentiate aldotersone producing adenoma (APA) and nonfunctional adenoma in essentital hypertension. The Hosmer–Lemeshow test showed no significant in training set (p = 0.43) and test set (p = 0.47) of radiomic model to differentiate aldotersone producing adenoma (APA) and nonfunctional adenoma in essentital hypertension, indicating that there was no departure from a perfect fit. While in the outcome prediction model, the Hosmer–Lemeshow test showed significant in both unenhanced CT (p < 0.05) and venous phase CT (p < 0.05) model, either in biomedical or clinical success prediction.

Figure 1. Calibration curves of models for nodule discrimination


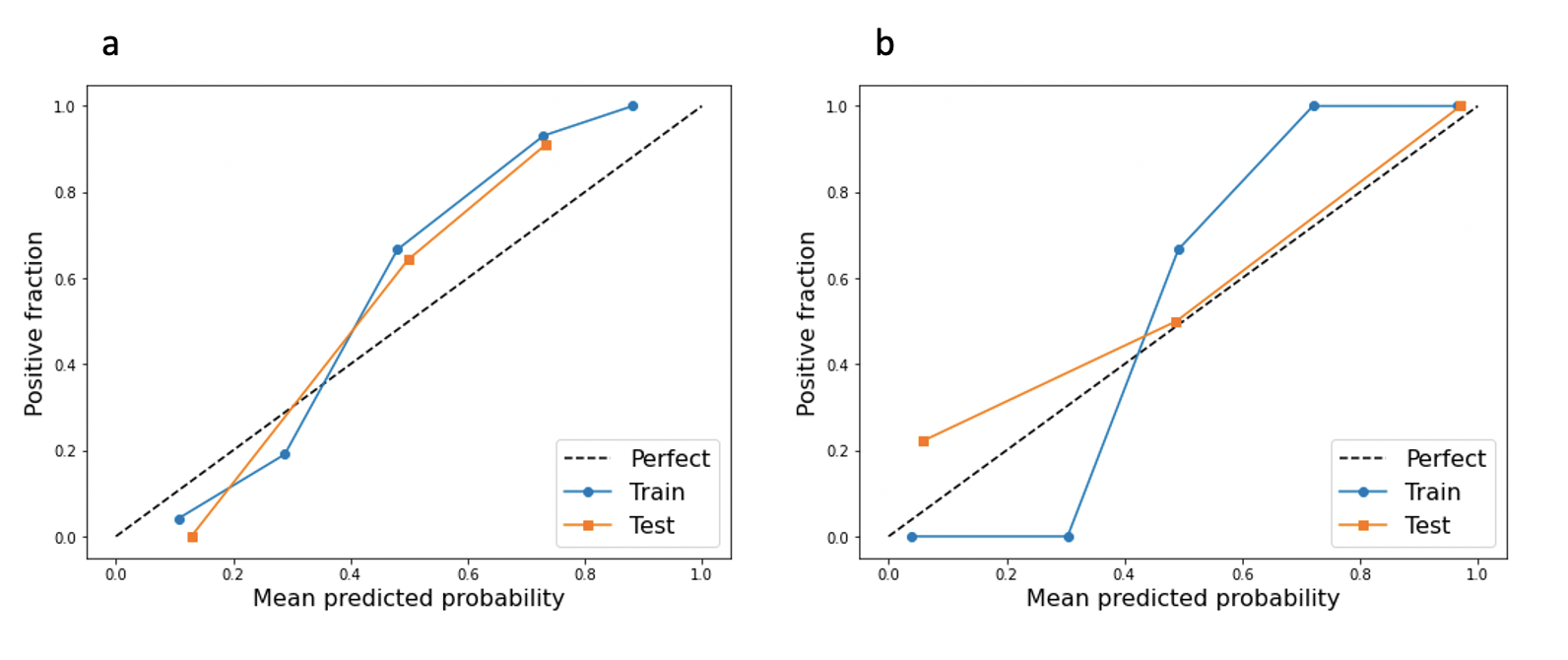


Calibration curves of differentiating APA and nonfunciontal adenoma in essential hypertension in unenhanced CT (a) and venous phase CT (b)

Figure 2. Calibration curves of models for outcome prediction


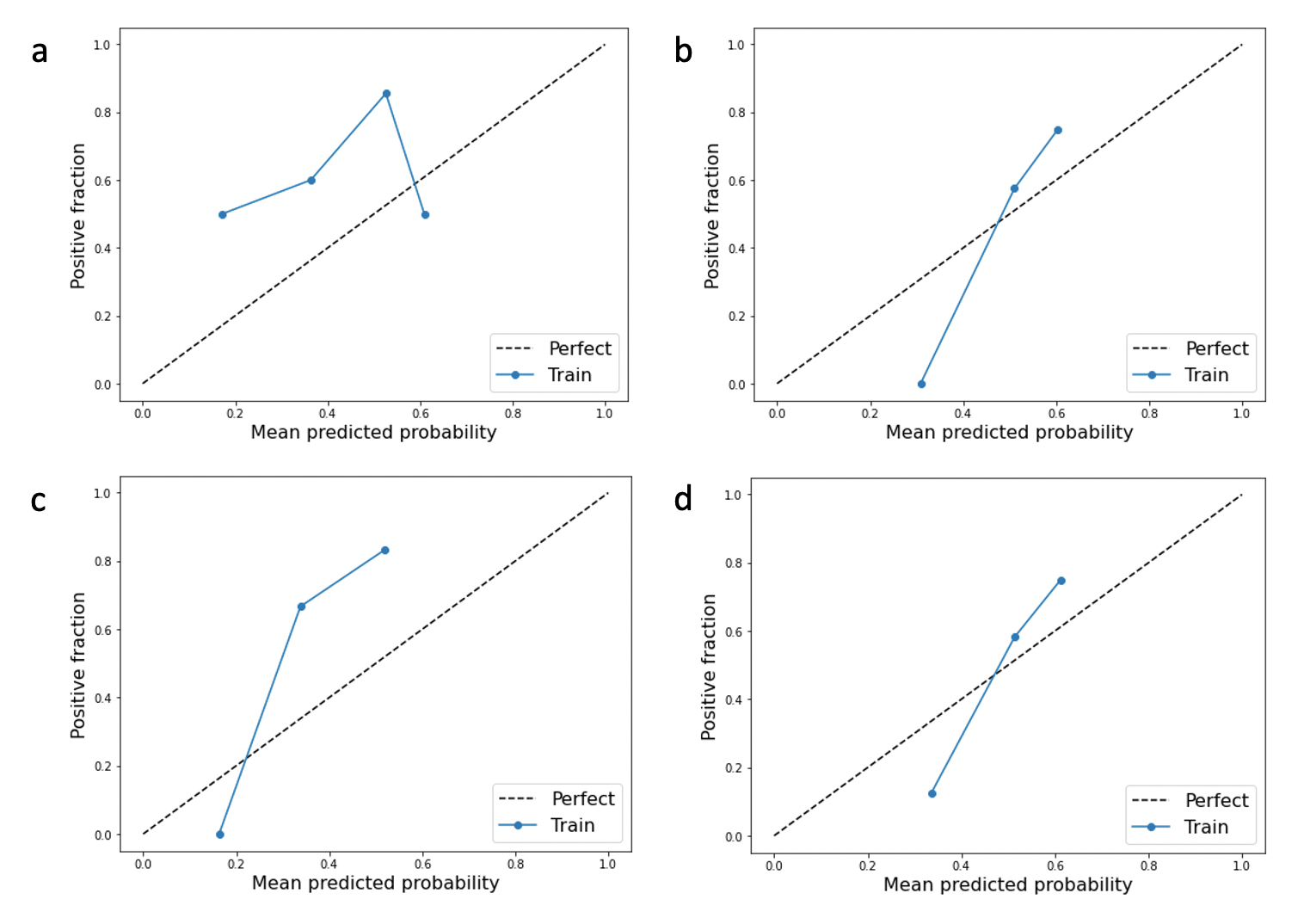


Calibration curves of outcome prediction of APA after adrenalectomy in unenhanced CT (a: biomedical success; b: clinical success) and venous CT (c: biomedical success; d: clinical success)
